# Tuning research competences for Bologna three cycles in medicine: report of a MEDINE2 European consensus survey

**DOI:** 10.1007/s40037-013-0066-z

**Published:** 2013-07-09

**Authors:** Richard Marz, Friedo W. Dekker, Chris Van Schravendijk, Siun O’Flynn, Michael T. Ross

**Affiliations:** 1 Department of Medical Education, Medical University of Vienna, DEMAW, Spitalgasse 23, 1090 Vienna, Austria; 2 Department of Clinical Epidemiology, Leiden University Medical Center, Leiden, the Netherlands; 3 Diabetes Research Center, Vrije Universiteit Brussel, Brussels, Belgium; 4 Medical Education Unit, School of Medicine, University College Cork, Cork, Ireland; 5 Centre for Medical Education, University of Edinburgh, Edinburgh, UK

**Keywords:** Tuning, Research competences, Medical curricula, Learning outcomes, Bologna process

## Abstract

Medical curricula, like healthcare systems and medical practice, have a strong cultural component and vary considerably between countries. Increasing mobility of medical graduates, and increasing pressure to ensure they are all fit for practice, have highlighted an urgent need to establish common ground in learning outcomes at all stages of training. A research-based approach, developed by the Tuning project, was used previously by the MEDINE Thematic Network to gain consensus on core learning outcomes/competences for primary medical degrees (www.tuning-medicine.com), but no consensus was reached for learning outcomes relating to research. As part of MEDINE2, a focussed Tuning project was undertaken to explore opinions on more detailed core learning outcomes in research for all three Bologna cycles (Bachelor, Master, and Doctor). Responses from 417 stakeholders, representing 29 European and 13 non-European countries, revealed a relatively high degree of consensus. The findings strongly suggest that these stakeholders think that learning outcomes related both to ‘using research’ and ‘doing research’ should be core components of medical curricula in Europe. The challenge now, however, is to promote further local and international discussion on these issues, and to find ways of achieving these competences within the context of already crowded medical curricula.

## Introduction

Research has been the driving force of many stunning advances in medicine over the last 100 years, and research is likely to substantially contribute to future developments in the field. Such statements are not likely to elicit much controversy—either from the medical academic community or from the public at large. Some medical curricula are internationally renowned for fostering an understanding of scientific method and for offering excellent research opportunities to students [1]. However, there are challenges in relation to the provision of such opportunities within already crowded medical curricula, and there are cultural and contextual variations in the curricular time and emphasis devoted to this.

The Bologna process now requires all European Higher Education institutions to adopt a three-cycle system of Bachelor, Master and Doctor degrees, with each ‘cycle’ lasting ~3 years [2]. Although some aspects of this remain controversial, it is generally accepted that the Master of Medicine equates to the primary medical degree (sometimes referred to as MD or MBBS), and the Doctor of Medicine to a higher degree (PhD or professional doctorate). It is also accepted that postgraduate speciality training (Residency) for hospital or general practice is much more advanced than the Master of Medicine, but is quite distinct from the Doctor of Medicine as it is clinically focused, usually situated outside the context of Higher Education, and often has no research component [3, 4]. Increasingly the content to be learned at each stage is being defined in terms of the intended learning outcomes of the curriculum, or the competences which graduates will possess on successful completion [4].

The primary purpose of an undergraduate medical curriculum (Bologna first and second cycles together) is to train doctors who are fit for practice, and consequently clinical competence often assumes primacy over other competing factors. Curricular time is precious, and as ‘clinical competence’ takes on an ever-wider meaning, additional learning outcomes in communication, cultural understanding, behavioural, attitudinal, leadership, management, teaching, and many other areas are also becoming essential. This increases the risk that learning outcomes pertaining to research may not be prioritized.

Many arguments have been advanced to support the position that all medical students should learn about research, ranging from developing competences which support core clinical activities such as critical reasoning and applying evidence-based medicine in patient care, to preparing and enabling students to enter into real research activities should they so choose. Research competences can be broadly categorized into one of three groups: ‘generic’ competences, those related to ‘using research’, and those related to ‘doing research’ [5]. ‘Generic’ competences, such as the ability to synthesize findings, and to draw conclusions from findings, are important in many areas of medical practice, including research. ‘Using research’ competences, such as the ability to define and carry out an appropriate literature search, and to critically appraise research evidence, are typically also considered important for most areas of medical practice. Many modern curricula have attempted to accommodate students who gravitate towards research activities with a structure of core and optional components, and there is an emergent evidence base relating to this approach [6–8]. Whether all medical graduates need to demonstrate competences related to ‘doing research’, such as the ability to formulate a research question as a hypothesis and to analyze research data remains, however, controversial [9, 10].

Students and academics may have different motivations in relation to research competences, at different stages in the three cycles of medical education. It may, for example, be challenging for a student during the Bachelor cycle to determine whether the ability to present and explain research results to peers would be important, whilst faculty are likely to have a different perspective.

The time available to obtain a meaningful research experience can be short. Whilst medical graduates may further develop their research competences by undertaking third cycle (PhD) degrees, many academics are concerned about inspiring and enabling research for all students in the first two cycles of undergraduate medical education. This would also seem to be a pertinent concern in an era where there has been significant investment in career pathways both within Europe and elsewhere to promote recruitment of medical physician-scientists [11, 12]. How can academics interest medical graduates in research and ensure they are sufficiently competent to undertake a PhD degree if the foundations for this are not laid in the first two cycles?

A study undertaken for the academic year 2005/2006 as part of the EU-funded MEDINE Thematic network [13] surveyed EU medical faculties and universities regarding the research component of their medical curricula [14]. It identified a wide disparity among countries and institutions in the content and structures for covering research competences (first and second cycle), with writing and defending a thesis being a formal second cycle (primary medical degree) requirement in around half the programmes. For instance, all medical students in the Netherlands are required to undertake a research project during the second cycle of at least 4–6 months (full time) duration, whilst in many other countries doing a research project is much shorter, optional, or only mandatory in certain medical schools. The structural gap widened even more when looking at third cycle (PhD) and professional education requirements of EU countries [15]. A further study undertaken by the MEDINE Thematic Network sought to gain consensus on core learning outcomes for primary medical degrees across Europe, using an established ‘Tuning’ methodology [4, 16]. No consensus was identified regarding the importance of learning outcomes relating to research in that study, although it was postulated at the time that this was at least in part due to the outcomes formulated and surveyed having been too broad and open to interpretation.

Thus, it was already known that there was considerable structural variation in EU-Europe with regard to whether and how much research training should be part of the medical curriculum [14]. However, it was not known to what extent consensus on core learning outcomes might be achievable if stakeholders were asked to give their opinions on a set of more detailed learning outcomes related to research. The current study aimed to identify consensus on which, if any, research competences all graduates of a medical curriculum should have achieved on successful completion of a Bachelor, Master, and Doctor of Medicine degree in Europe. This study constitutes a major output from Work Package 7 (‘Integration of the Research Component in European Medical Education’) of the MEDINE2 Erasmus Thematic Network for Medical Education in Europe 2009–2013, coordinated by the University of Edinburgh and supported by funding from the Life Long Learning Programme of the European Commission [17].

## Methods

The methodology employed in the original Tuning (Medicine) project was adopted as far as possible [18], although further work was required to develop a draft set of competences/learning outcomes in research. In addition to expanding on the learning outcomes in research that had been used in the original Tuning (Medicine) project, other existing literature related to research outcomes from various related fields was reviewed. The distinction between competences relating to using and doing research was also found to be useful in reviewing and synthesizing this literature.

The study therefore consisted of:

1. Review by the project group of existing frameworks of research competences, including those of senior researchers, and learning outcomes in medicine in Europe and further afield [4, 19–26].
2. Development of a draft framework of research competences in medicine in a 1 day work-package meeting in Madrid in September 2010, involving 14 participants from 11 European countries. This provisional framework was sequentially refined through feedback obtained following presentations and during informal consultations. An online discussion among the project group was then used to formulate 31 competences.
3. Based on discussions with all authors of the manuscript, each competence was assigned to one of the three categories: ‘generic’, ‘using research’, and ‘doing research’, and allocated a unique identifier (C1–C31, Table 1). Respondents were unaware of this classification.

**Table 1:** Research competences, ranked by the percentage of respondents considering a competency ‘not important’ at the end of cycle 2

Item I.D.	Rank	Graduates will have the ability to …	Generic	Using	Doing	% Rated ‘not important’ by …			Leik measure of consensus for …		
						End of 1st cycle	End of 2nd cycle	End of 3rd cycle	End of 1st cycle	End of 2nd cycle	End of 3rd cycle
C31	1	Use computers effectively	X			1.9	0.8	0.5	0.51	0.64	0.79
C2	2	Define and carry out an appropriate literature search		X		8.4	1.0	0.5	0.62	0.67	0.89
C16	3	Synthesize findings and draw conclusions	X			21.1	3.1	0.8	0.66	0.67	0.84
C9	4	Recognize, discuss and prevent scientific misconduct	X			21.1	3.3	0.5	0.58	0.61	0.78
C11	5	Maintain confidentiality and protect data	X			11.8	3.3	0.5	0.41	0.53	0.80
C30	6	Write and speak in English	X			10.3	3.5	1.1	0.42	0.55	0.77
C4	7	Critically appraise published medical literature including observational, interventional, and meta analysis using established critical appraisal guidelines		X		34.2	4.2	0.3	0.64	0.63	0.78
C14	8	Analyze research findings (qualitative or quantitative data)			X	23.6	4.4	1.0	0.71	0.65	0.83
C8	9	Apply ethical principles and analysis to research, seeking ethical approval where appropriate			X	21.8	4.6	1.3	0.49	0.55	0.79
C3	10	Keep track of the pertinent scientific literature		X		33.1	4.8	0.8	0.60	0.62	0.80
C15	11	Select and carry out appropriate statistical tests and interpret the results			X	31.0	5.9	2.1	0.65	0.62	0.74
C1	12	Formulate a research question as a hypothesis and design experiments to test it			X	37.7	6.6	0.8	0.61	0.60	0.82
C21	13	Present research results obtained by others, e.g. in a journal club		X		28.9	6.9	0.8	0.66	0.60	0.64
C19	14	Present research results to peers, e.g. in scientific meetings			X	36.6	8.1	1.0	0.58	0.58	0.78
C26	15	Contribute effectively to a research team			X	35.2	8.6	1.3	0.62	0.54	0.67
C10	16	Obtain and record informed consent for participation in research	X			31.9	8.7	1.0	0.50	0.52	0.70
C7	17	Choose the appropriate qualitative or quantitative research method			X	47.4	9.9	1.2	0.60	0.55	0.72
C28	18	Communicate scientific findings to lay people	X			37.1	12.2	2.4	0.60	0.51	0.57
C13	19	Carry out research on medical practice			X	52.3	12.2	2.3	0.59	0.53	0.57
C18	20	Disseminate research findings		X		50.8	15.6	2.6	0.58	0.52	0.60
C17	21	Propose and carry out the next step in a research project			X	56.2	15.7	1.8	0.64	0.55	0.64
C12	22	Apply national and European law to research			X	41.5	16.1	3.1	0.49	0.44	0.53
C20	23	Write a scientific paper suitable for publication			X	59.7	17.8	1.8	0.65	0.50	0.79
C6	24	Carry out laboratory procedures			X	40.5	19.9	9.7	0.64	0.55	0.45
C5	25	Design a research project, including project planning and allocation of resources			X	63.6	22.4	2.7	0.67	0.59	0.64
C22	26	Contribute to research-funding proposals			X	68.7	26.5	3.5	0.74	0.59	0.54
C29	27	Critically evaluate research proposals		X		67.3	31.5	3.8	0.72	0.56	0.55
C23	28	Write research-funding proposals			X	77.9	36.5	4.0	0.82	0.59	0.53
C25	29	Supervise laboratory technicians			X	83.8	48.7	11.9	0.85	0.53	0.47
C24	30	Supervise research students			X	87.4	54.7	6.6	0.87	0.57	0.54
C27	31	Lead a research team			X	86.0	59.4	14.0	0.87	0.61	0.44
4. This list of 31 research competences was developed into a detailed questionnaire using an online survey instrument, [27] ordered by their unique identifiers C1–C31. As in the original Tuning (Medicine) study, academics, students, graduates and employers were asked to indicate how important they thought it was for graduates to have achieved each competence by the end of each of the three Bologna cycles [4]. A 4-point Likert scale (Not Important/Important/Very Important/Essential) was used (Fig. 1). Demographic data were also recorded.

**Fig. 1:**
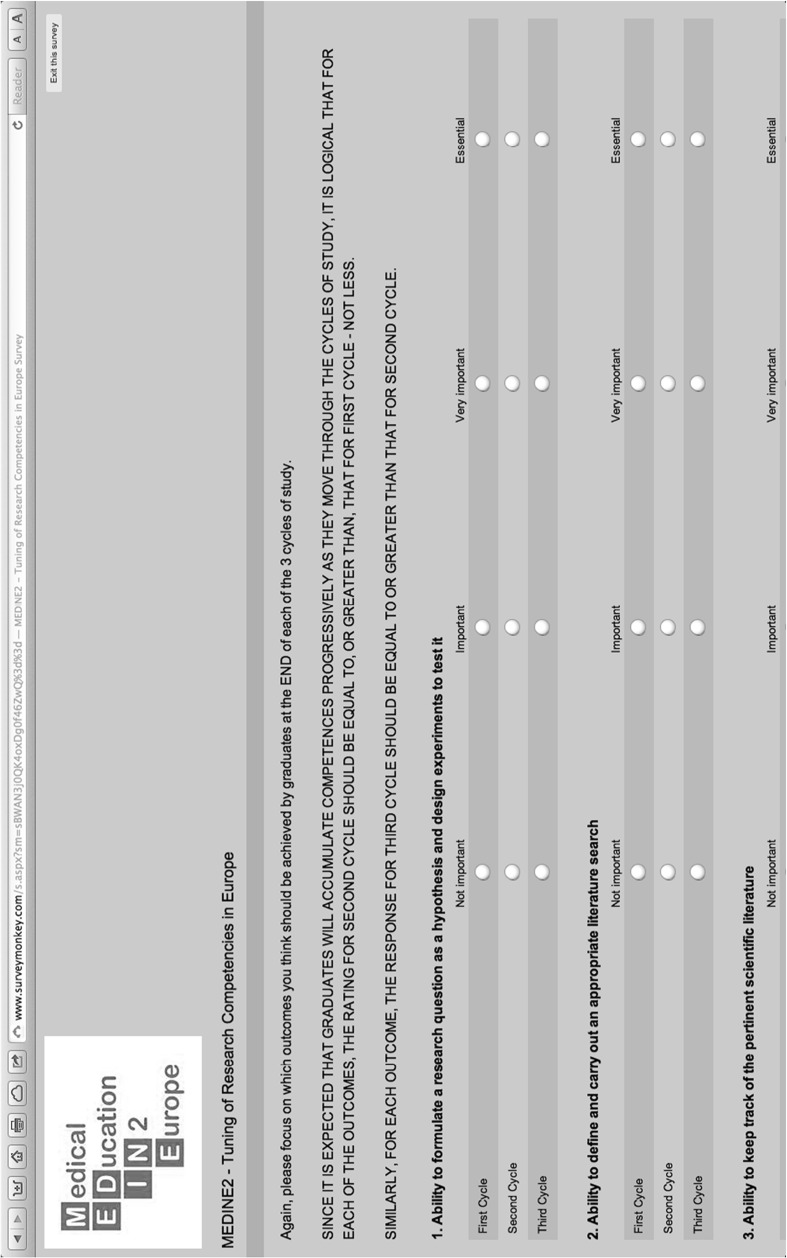
Example of questions (C1–C3) from SurveyMonkey
5. The online questionnaire was implemented with Survey Monkey in English only, and was available online from June 2011 until May 2012. The survey was accessible via the project home page and was publicized using various networks with the aim of acquiring mainly respondents from EU countries. Response demographics were regularly analyzed whilst the survey was live, and targeted reminders were sent to key contacts in under-represented countries and respondent groups.
6. Demographic data were collated, and learning outcomes were ranked in order of perceived importance. For data analysis and interpretation purposes, responses were dichotomized into ‘not important’ versus ‘important’ (the sum of Important + Very Important + Essential). Differences between academics and students were explored with χ^2^ tests. In order to test the degree of agreement for each individual item, the Leik measure of ordinal consensus was calculated for each item and group of items [28]. This uses the frequency with which each option is endorsed to assess how much consensus there is for the ranking of each item. Higher numbers always imply a greater degree of consensus. As a general guide, values below 0.20 are poor, values between 0.21 and 0.40 are fair, values between 0.41 and 0.60 are moderate, values between 0.61 and 0.80 are substantial and values above 0.80 are good. Intra-class correlation coefficients were calculated for any sub-groups large enough to skew the overall rankings, and mean and standard deviations were compared with and without responses from these groups.
7. All data and analyses were evaluated and interpreted in a project group workshop. The results were presented, discussed, and approved by the entire MEDINE2 Thematic Network at the final Annual General Meeting in Edinburgh in September 2012. The final report and framework of intended learning outcomes/graduate competences will be presented to the European Commission in late 2013.

## Results

### Demographics

There were 417 responses to the survey, from stakeholders in 29 European and 13 non-European countries. All EU countries were represented except Bulgaria, Cyprus, Estonia, Latvia and Luxembourg. Sixty-eight respondents did not specify the country in which they were located, and 26 indicated they were in the USA, Africa, Asia or Australia. The largest number of responses came from Austria (55), Spain (39), Finland [35], Turkey [33], Germany [20] and the Netherlands [19], although none of these subgroups seemed to skew the overall rankings. Two-thirds of respondents were academics involved in delivering undergraduate medical education (including 88 who considered themselves responsible for a medical curriculum in 23 different European countries), and 28 % of respondents were medical students.

### Consensus on learning outcomes

Detailed results of the survey are presented in Table 1. For each of the 31 competences the percentage of respondents indicating a specific item as ‘not important’ at the end of all three cycles is given. The responses are ranked according to the outcome at the end of the 2nd cycle (primary medical degree). For example, C31 was considered ‘not important’ by 0.8 % and thus considered ‘important’ by the remaining 99.2 %. The Leik measure of consensus and the category to which each competence was assigned are also listed in Table 1.

The degree of consensus varied across cycles. Some items began with a high degree of consensus in cycle 1 which declined significantly by cycle 3, or vice versa. For example, when asked to rank the importance of C8 (Apply ethical principles and analysis to research, seeking ethical approval where appropriate), there was only moderate consensus on the importance of this for the first cycle, but by the third cycle the consensus was borderline between substantial and good. On the other hand, when asked to rank the importance of C24 (Supervise research students), the consensus was initially good—they agreed that it was ‘not important’—but by the third cycle the consensus had declined to only moderate. It is of interest to note how uniformly respondents answered in each cycle. In general, the consensus tended to vary between substantial and good. Some small variations between the responses of academics and students were also identified and are described below.

### Bachelor of medicine (Bologna first cycle)

At the end of first cycle, only four items were considered ‘not important’ by <15 % of respondents, of which three were ‘generic’ competences: C11 (Maintain confidentiality and protect data), C30 (Write and speak in English), and C31 (Use computers effectively). Only one item related to ‘using research’ was considered ‘important’ at this stage by more than 90 % of respondents: C2 (Define and carry out an appropriate literature search). There was a statistically significant difference in the greater importance students assigned to five items (C13, C22, C23, C27, and C29), at the end of the first cycle compared with academics.

### Primary medical degree (Bologna second cycle)

For ten competences, <5 % of respondents considered them ‘not important’ to be obtained at the end of the second cycle. For a further nine competences, 5–15 % considered them ‘not important’ (Table 1). Seven of these top-19 competences were classified as ‘generic’, four as ‘using research’, and eight as ‘doing research’. There was a statistically significant difference in the importance students assigned to five items (C13, C24, C27, C28, and C29), at the end of the second cycle—each of these items were considered more important by students than by academics. Remarkably C13 (Carry out research on medical practice) was considered ‘not important’ by 14.5 % of academics, but only by 6.1 % of students, suggesting that students may view doing research as more central to their professional life than their academic teachers.

### Doctor of medicine (Bologna third cycle)

At the end of the third cycle, 27 items were considered ‘not important’ by <5 %, and thus ‘important’ by more than 95 %, of respondents. The remaining 4 items: C6 (Carry out laboratory procedures), C25 (Supervise laboratory technicians), C24 (Supervise research students), and C27 (Lead a research team) were regarded by many as being necessary competences with only 5–15 % assigning a low importance to them. Two items: C17 (Propose and carry out the next step in a research project) and C20 (Write a scientific paper suitable for publication) showed a sizable increase in their perceived importance between the end of the 2nd and 3rd cycle. The only significant difference (*p* = 0.011) between academics and students was found for C27 (Lead a research team): 18 versus 7 % respectively considered this ‘not important’ for the end of the third cycle, possibly indicating that academics assign relatively more significance to this skill.

## Discussion and conclusions

The survey results reveal a surprising amount of consensus between stakeholders about core competences relating to research for each of the three Bologna cycles. Particularly interesting is that the ability to do research was considered at least as important as being able to use research, especially at the end of the second cycle. It is perhaps not surprising that students and academics may assign different importance to learning outcomes related to research. One could speculate that faculty who appreciate the importance and benefits of research output would prioritize it. However, we were interested to note that in many cases in relation to doing research students assigned more importance to these outcomes than staff. It is beyond the scope of the current research to establish why this is the case, but others have documented a range of motivations which may influence students in this regard [6–8, 29]. We may speculate, however, that the immediacy of seeking postgraduate training places and the recognition that research competences and output will enhance the available opportunities are likely to have some influence [30], as there is increasing evidence that students actively seek research opportunities for this purpose [7, 29, 30].

Students also assigned more importance to C13 (Carry out research on medical practice). Clearly undergraduates are and seek to be research active in the first and second cycle also in non-laboratory settings. This may require a transition in emphasis in some medical curricula [31]. Facilitating and supporting non-laboratory medical research can also present additional inherent challenges for ethical reasons, supervision and governance [32]. It is possible that non-clinical academics would assign less importance to this competence, due to their frame of reference, than would clinical academics, but this was not specifically explored in the current research.

### Limitations of this research

The findings of participant surveys are always subject to interpretation, despite every effort to make them clear and precise. For example, it is possible that outcome C6 (Carry out laboratory procedures), was interpreted by some respondents as routine clinical laboratory tests rather than as laboratory procedures for the purpose of research. As the survey was undertaken in English, it is possible that English-speaking respondents are over-represented. Translations introduce new problems with interpretation, however, and in other Tuning surveys that have been translated, the majority of respondents have in any case chosen to respond in English. We were somewhat surprised that 60 % of respondents rated the competence of writing a research proposal as important at the end of the second cycle. However, it is possible that respondents also interpreted this differently as research proposals vary considerably in scope and depth.

### Reflection on key findings

There are several markers of academic rigour in the current research. For one, participants were asked to identify themselves and to supply their email address, which more than 85 % did, allowing plausibility checks. Outcomes that define increasing independence as a researcher, such as C17 (Propose and carry out the next step in a research project), were considered less important for the second than for the third cycle. C27 (Lead a research team) was considered by 14 % as ‘not important’ for the third cycle and thus was not considered a consensus outcome—which, to members of the MEDINE2 Network, gave it face validity. Internal consistency like this supports the reliability of these findings.

Given the huge diversity of the structure of third cycle programmes within Europe [13], the survey data which pertain to intended learning outcomes/graduate competences, show an impressive degree of consensus. These findings do not in any way undermine the lack of consensus achieved with regard to research competences in the original Tuning (Medicine) project, but rather reflect that in the current study the learning outcomes, which stakeholders were asked to consider, were defined in more detail and were therefore probably more straightforward to rate with regard to importance. Similarly, future studies exploring consensus on the importance of these competences in more detail with different respondents may achieve different results.

Diversity in responses might therefore be considered to be mostly the consequence of different traditions as well as contextual, institutional, and national priorities, rather than fundamental disagreements. It could be argued that some diversity is inevitable, and indeed desirable. If we wish to produce doctors who can critically evaluate the evidence base and incorporate this analytical approach to the evaluation of their own practice, however, it would seem reasonable to expect a minimal essential level of research competence. Furthermore, research attainments are increasingly required to access postgraduate (after second cycle) clinical training, and therefore if mobility is to be promoted and achieved, consensus regarding research training at the various cycle stages is desirable.

Whilst this work will not lead to a European blueprint for three-cycle medical education with regard to research in the near future, it does suggest that significant consensus in relation to core learning outcomes can be achieved. It must be noted that there are also other initiatives, which attempt standardization in this area [3]. Further research is required to determine how these competences might be best achieved and demonstrated by students, and also to identify and share best practices across Europe and beyond.

What are the take-home messages for programmes leading to a primary medical degree? These findings suggest that there is support from students and academics that teaching research competences (to do research) should be part of all medical curricula in Europe. Most respondents agreed on 19 consensus competences in research for primary medical degrees, and we suggest that these be considered when planning an outcome-based medical curriculum. Those in the ‘generic’ and ‘using research’ categories are typically already part of many curricula [14], but there are still differences of opinion with regard to how some of these can be achieved, whether through ‘doing research’ or by other means. Prior to this research, however, it was not clear to what extent stakeholders consider that ‘doing research’ should be part of the European medical curriculum.

The survey asked respondents to consider the 3rd cycle (PhD) curricula, but not further postgraduate training for clinical specialist and general practice. The opportunities for medical graduates to pursue research at a postgraduate level vary by location and speciality and are not limited to the university environment, which puts additional demands on the research qualifications for 2nd cycle graduates. The results of the survey indicate, however, that there is support for a research orientation of medical curricula. Debate will continue as to whether research-enabled graduates are also better doctors clinically. If approaches are inconsistent across Europe, however, there is a risk that this will act as a barrier to mobility. Many postgraduate clinical training programmes producing hospital-based specialists require trainees to provide evidence of research activity. Increasingly research in the form of clinical audit is a basic requirement of competence assurance. In Ireland, for example, each registered practitioner must submit annually in writing a formal standards-based review of an aspect of their practice [33]. Also the Accreditation Council for Graduate Medical Education lists as a common requirement for the Accreditation of post-MD medical training programmes within the United States, that ‘The curriculum must advance residents’ knowledge of the basic principles of research, including how research is conducted, evaluated, explained to patients, and applied to patient care’ [34]. Applicants must usually have evidence of research output to access such programmes, and therefore research-enabling must have occurred in the second cycle to render them eligible to access research focused post-Masters training.

The question ‘what educational models cultivate the educated practitioner?’ has recently been revisited by Coles [35]. Building on the work of Stenhouse [36], three possible educational models: ‘product’, ‘process’, and ‘research’ are examined in some detail. While medical curricula must include all approaches to some extent, Coles argues that the research approach is the most appropriate in many instances, since it engages learners in researching their clinical practice [35]. This would suggest that there may be other advantages to learning even ‘generic’ competences through a research approach. Taken together with the findings of this survey, it would seem to support the view that students should obtain research competences by actually doing research as part of all medical curricula in Europe.

## Essentials

- It is becoming increasingly important to define and gain consensus on core competences for medical training across Europe.
- The previous Tuning Project (Medicine) achieved consensus on core competences for many areas of the primary medical (undergraduate) degree across Europe, except for those related to research.
- The practice of modern medicine depends on the application of evidence from medical research, and future medical research is required for on-going development in the field; however, opportunities to learn about research in European undergraduate medical curricula vary enormously.
- This study defined a set of detailed competences related to research and then, using an online stakeholder survey, achieved broad consensus on the importance of each for the Bachelor, Master and Doctor of Medicine across Europe.
- Competences related to doing research, as well as using research, were considered by most respondents to be important for the primary medical degree—supporting the view that some research experience should be included in all European undergraduate medical curricula.
